# Identification of *SRXN1* and *KRT6A* as Key Genes in Smoking-Related Non-Small-Cell Lung Cancer Through Bioinformatics and Functional Analyses

**DOI:** 10.3389/fonc.2021.810301

**Published:** 2022-01-05

**Authors:** Jiazhen Zhou, Guanqing Jiang, Enwu Xu, Jiaxin Zhou, Lili Liu, Qiaoyuan Yang

**Affiliations:** ^1^ The Institute for Chemical Carcinogenesis, School of Public Health, Guangzhou Medical University, Guangzhou, China; ^2^ Department of Thoracic Surgery, General Hospital of Southern Theater Command, People’s Liberation Army (PLA), Guangzhou, China; ^3^ Guangdong Provincial Key Laboratory of Occupational Disease Prevention and Treatment, Guangdong Province Hospital for Occupational Disease Prevention and Treatment, Guangzhou, China; ^4^ State Key Laboratory of Respiratory Disease, The First Affiliated Hospital of Guangzhou Medical University, Guangzhou, China

**Keywords:** lung cancer, non-small-cell lung cancer, smoking-related gene, *KRT6A*, *SRXN1*

## Abstract

**Background:**

Lung cancer is the leading cause of cancer-related mortality worldwide. Although cigarette smoking is an established risk factor for lung cancer, few reliable smoking-related biomarkers for non-small-cell lung cancer (NSCLC) are available. An improved understanding of these biomarkers would further the development of new biomarker-targeted therapies and lead to improvements in overall patient survival.

**Methods:**

We performed bioinformatic analysis to screened potential target genes, then quantitative PCR, western, siRNA, CCK-8, flow cytometry, tumorigenicity assays in nude mice were performed to validated the function.

**Results:**

In this study, we identified 83 smoking-related genes (SRGs) based on an integration analysis of two Gene Expression Omnibus (GEO) datasets, and 27 hub SRGs with potential carcinogenic effects by analyzing a dataset of smokers with NSCLC in The Cancer Genome Atlas (TCGA) database. A survival analysis revealed three genes with potential prognostic value, namely SRXN1, KRT6A and JAKMIP3. A univariate Cox analysis revealed significant associations of elevated SRXN1 and KRT6A expression with prognosis. A receiver operating characteristic (ROC) curve analysis indicated the high diagnostic value of SRXN1 and KRT6A for smoking and cancer. Quantitative PCR and western blotting validated the increased expression of SRXN1 and KRT6A mRNA and protein, respectively, in lung cancer cell lines and NSCLC tissues. In patients with NSCLC, SRXN1 and KRT6A expression was associated with the tumor–node–metastasis (TNM) stage, presence of metastasis, history of smoking and daily smoking consumption. Furthermore, inhibition of SRXN1 or KRT6A suppressed viability and enhanced apoptosis in the A549 human lung carcinoma cell line. Tumorigenicity assays in nude mice confirmed that the siRNA-mediated downregulation of SRXN1 and KRT6A expression inhibited tumor growth *in vivo*.

**Conclusions:**

In summary, SRXN1 and KRT6A act as oncogenes in NSCLC and might be potential biomarkers of smoking exposure and the early diagnosis and prognosis of NSCLC in smokers, which is vital for lung cancer therapy.

## Introduction

Lung cancer is currently a leading cause of death in both men and women worldwide, and the combined lung cancer-related death rate exceeds that of the three most common incident cancers (colon, breast and pancreatic) combined. NSCLC accounts for approximately 84% of lung cancer diagnoses; at diagnosis, approximately 57% of these tumors have metastasized, 22% have spread to regional lymph nodes and 16% remain localized (1). Clinically, only a small proportion of NSCLC patients are diagnosed at an early stage (stage І or П), when the tumor can be treated by surgical resection (2).

The status of lung cancer as one of the most common causes of cancer death persists, despite an understanding of the major etiology. Epidemiological studies have identified cigarette smoking as the most important risk factor for lung cancer, and 80% and 50% of lung cancers in male and female patients, respectively, are associated with cigarette smoking. Particularly, smoking is associated with a 5- to 10-fold increase in the risk of lung cancer, and this relationship exhibits a clear dose–response pattern (3). In other words, an increase in the number of smoking years or the number of packs smoked per day increases the degree of lung cancer risk (4). Additionally, exposure to environmental tobacco smoke increases the lung cancer risk by approximately 20% among non-smokers (3). In summary, the vast majority of lung cancers occur in people aged >50 years with a history of cigarette smoking (5), whereas only 10–15% of cases involve non-smokers (6). An understanding of the epidemiology and causal factors of lung cancer can provide an additional foundation for disease prevention.

Although most countries have long made efforts to reduce tobacco consumption and exposure, the 2015 global report on trends in the prevalence of tobacco smoking by the World Health Organization revealed that there are 1.1 billion adult smokers and at least 367 million smokeless tobacco users worldwide, and that more than 6 million people die from tobacco-related causes every year (7). Today, passive exposure to tobacco smoke (i.e., passive smoking) is recognized as an important environmental risk factor for the development of lung cancer, asthma and fetal growth (8–10). For example, one meta-analysis and comprehensive review determined that the relative risk of developing lung cancer from passive smoking ranged from 1.14 to 5.20 among people who had never smoked but lived with a smoker (11). According to the U.S. Surgeon General, living with a smoker can increase a non-smoker’s risk of developing lung cancer by approximately 20–30% (12). In China, approximately 16% of lung cancer cases among never-smokers are potentially attributable to passive smoking (13).

Globally, smoking is a major public health problem, and exposure to environmental tobacco smoke (ETS) affects both smokers and passive smokers. ETS is known as an important source of multiple pollutants, and 73 of the more than 5,000 compounds identified in cigarette smoke are considered carcinogenic to either laboratory animals or humans by the International Agency for Research on Cancer (14, 15). Although many components of tobacco smoke contribute to lung cancer, nitrosamine 4-(methylnitrosamino)-1-(3-pyridyl)-1-butanone (NNK) is a key ingredient with a major role in carcinogenesis (16). Studies on tobacco smoke have confirmed NNK as the most potent lung carcinogen and, to date, it is the only tobacco carcinogen that systemically induces lung tumors in rats, mice and hamsters (i.e., three of the most common rodent models) (17).

The relevance of NNK in triggering lung tumorigenesis has been described consistently and confirmed in numerous studies. It not only mutates or activates oncogenes and tumor suppressor genes such as *Adrb2*, *Kras*, *Tp53* and *Txa2* (18–21), but also induces hypermethylation of the promoters of multiple tumor suppressor genes. Specifically, *DNMT1* is overexpressed in the lung, and the resulting hypermethylation reduces the expression of tumor suppressor genes such as *IGFBP-3*, *CDKN2A*, death-associated protein kinase 1 (*Dapk1*), retinoic acid receptor β (*Rar-β*) and runt-related transcription factor 3 (*Runx*) (22–25). Another study suggested that NNK stimulated the Erk signaling pathway and induced cell transformation and proliferation *via* the epidermal growth factor receptor signaling pathway (26). Furthermore, NNK can significantly stimulate thromboxane synthase activity and enhance LUSC generation, as indicated by the upregulated expression of CD133 and ALDH1A1 and increases in the tumor sphere number and size (27). NNK also promotes the expression of LCSC-related molecules, including β-catenin and Nanog (27). Therefore, NNK, as the dominant carcinogen in tobacco smoke, is the key factor in smoking-related lung cancer, and it is necessary to disrupt the key mechanisms linking its effects with lung cancer development.

This study aimed to identify novel biomarkers for the early diagnosis of smoking-related NSCLC. To address this issue, we extracted data from GEO and TCGA databases and compared the differentially expressed genes (DEGs) between lung cancer and normal tissues from smoking and non-smoking subjects. Notably, we identified *SRXN1* and *KRT6A* as pivotal smoking-related genes (SRGs) with important roles in lung cancer carcinogenesis through subsequent experimental validation *in vitro* and *in vivo*.

## Materials and Methods

### Data Processing

The raw data and clinical information of healthy participants were downloaded from the GEO (https://www.ncbi.nlm.nih.gov/geo/). Gene expression profiling of the GSE18385 (28) and GSE76324 (29) datasets was conducted using the GPL570 platform (Affymetrix Human Genome U133 plus 2.0 Array). The GSE18385 series comprised samples from 72 never-smokers and 89 smokers, and the GSE76324 series comprised samples from 97 never-smokers and 120 smokers. Samples with >20% missing expression values were excluded. Finally, 60 and 84 samples from never-smokers and smokers, respectively, in the GSE18385 dataset and 83 and 76 samples from never-smokers and smokers, respectively, in the GSE76324 dataset were enrolled. The processing of mRNA-seq data was based on the normalized chip values from the pre-processed data that were subjected to log2 transformation and submitted for analysis.

The raw data and clinical information of currently smoking patients with NSCLC were downloaded from TCGA (https://cancergenome.nih.gov). **Supplementary Table S1** summarizes the clinical characteristics of 256 current smoking patients with NSCLC up to September 2019. Of these patients, 226 patients whose clinical files unambiguously indicated the smoking status were selected based on the described neoplastic and histological information. Patients with a previous history of cancer and whose tissues were fixed with formalin and embedded in paraffin were excluded. The samples with >20% missing expression values were excluded. The processing of mRNA-seq data was based on the normalized count reads from pre-processed data subjected to log2 transformation after adding a 0.5 pseudocount.

### Differential Gene Expression Analysis and Pathway Enrichment Analysis

After data pre-processing, the differential gene expression analysis was conducted by using the limma and DESeq2 packages in R (v. 3.6; R Project for Statistical Computing, Vienna, Austria) (Love et al., 2014). An absolute log2 fold change value >1 and cut-off value of 0.05 were applied for the both raw *P*-value and Benjamini–Hochberg (BH) adjusted *P*-value (Benjamini and Hochberg, 1995). Kyoto Encyclopedia of Genes and Genomes (KEGG) pathway analyses were conducted using the DAVID platform (https://david.ncifcrf.gov/). KEGG pathways with *P*-values <0.05 were selected for bar chart in R.

### Survival Analysis and Cox Regression Analysis

For gene expression and patient survival analyses, the lung cancer patients were classified into high and low gene expression groups according to the median expression value. We used the survival and survMine tools on the R platform to conduct the survival analysis and generate the Kaplan–Meier (KM) survival plots. We additionally performed a univariate Cox regression analysis and log-odd tests for genes in the context of significant difference in patient survival in the high and low expression group. Hazard ratios (HRs) >1 and <1 indicated low and high rates of survival, respectively, among patients with high expression of the target gene.

### Logistic Regression Analysis

We conducted a logistic regression using the lm function in R to classify the samples from smokers and never-smokers based on the gene expression data. Similarly, we used the same way to classify the tumor and normal samples on the basis of their gene expression data. The R package ROCR was used to assess the logistic regression performance, plot receiver operating characteristic (ROC) curves and calculate the areas under the curves (AUC).

### Patients and Tissue Samples

Seventy-five pairs of NSCLC and normal adjacent non-tumor tissue samples were obtained by surgical resection between August 2017 and June 2019 at the General Hospital of Southern Theater Command (Guangzhou, China). Patients who received preoperative treatments such as radiation or chemotherapy were excluded. The paired adjacent non-tumor tissues were sampled at a distance of 3 cm from the tumor. Both the cancer tissues and matched normal tissues were histologically confirmed. All tissues were snap-frozen in liquid nitrogen and stored at -80°C. The clinicopathological characteristics of the 75 NSCLC patients, including sex, age, smoking history, daily smoking consumption, histology, TNM stage, tumor size, lymph node stage and distant metastasis status, are summarized in **Supplementary Table S2**. All clinicopathological data were obtained from the patients’ clinical and pathologic records.

The study protocol was approved by the Research Ethics Committee of Guangzhou Medical University and the General Hospital of Southern Theater Command. Written informed consent was obtained from all patients.

### Cell Lines and Cell Culture

The human bronchial epithelial cell line Beas-2B and the NSCLC cell lines A549 and 95D were obtained from the Chinese Academy of Sciences’ typical culture preservation committee cell bank (Shanghai, China). Beas-2B cells were induced to undergo malignant transformation by exposure to NNK in our laboratory as previous report (designated as BEAS-2B-NNK) (30). Beas-2B and BEAS-2B-NNK cells were cultured in bronchial epithelial basal medium (BEBM, Clonetics/Lonza, Basel, Switzerland) supplemented with SingleQuots (Clonetics). A549 and 95D cells were cultured respectively in F-12 Kaighn’s Modification (HyClone, Logan, UT, USA) and RPMI-1640 (HyClone) media supplemented with 10% fetal bovine serum (FBS; Gibco, Thermo Fisher Scientific, Inc., Chicago, IL, USA). All cells were cultured at 37°C in a humidified incubator in an atmosphere of 95% air and 5% CO_2_.

### RNA Extraction and Quantitative Real-Time PCR Assay

Total RNA was extracted using TRIzol reagent (Invitrogen, Carlsbad, CA, USA) according to the manufacturer’s protocol. Next, cDNA was prepared from total RNA *via* reverse-transcription (RT), using the GoScript Reverse Transcription System (Promega, Madison, WI, USA) according to the manufacturer’s instructions. Quantitative real-time PCR (qPCR) was performed on an Applied Biosystems™ 7500 Fast Dx Real-Time PCR Instrument (Foster City, CA, USA) with GoTaq qPCR Master Mix (Promega, Madison, WI, USA) according to the following program: denaturation, 95°C for 10 min and 36 cycles of denaturation at 95°C for 25 s, annealing at 60°C for 1 min and extension at 72°C for 30 s. The expression data were calculated using the 2^-ΔΔCt^ method. The results were normalized to the expression of the housekeeping gene, *GAPDH*. All primers were synthesized by Invitrogen (Shanghai, China). The primer sequences for RT-qPCR were shown in **Table 1**.

**Table 1:** Primer sequences used in RT-qPCR.

Gene	Primer sequences
SRXN1	5′-AAGGGTGTACCTGGGAGCAT-3′ (F)
	5′-CGCCAGGTGCAAAGAGAATG-3′ (R)
KRT6A	5′-CCAAGGCAGACACTCTCACA-3′ (F)
	5′-TCCTCATATTGGGCCTTGAC-3′ (R)
GAPDH	5′-ACAGTCAGCCGCATCTTCTT-3′ (F)
	5′-GACTCCGACCTTCACCTTCC-3′ (R)

### Western Blotting

Cells were harvested, lysed with RIPA buffer (Beyotime, Jiangsu, China) on ice for 30 min and centrifuged at 14,000 rpm and 4°C for 20 min. The total protein concentrations in the lysates were quantified using a BCA protein assay kit (Beyotime). The proteins were then separated by sodium dodecyl sulfate–polyacrylamide gel electrophoresis (SDS-PAGE) and transferred to PVDF membranes (Millipore, Burlington, MA, USA). The membranes were blocked in buffer containing 5% skim milk and 0.1% Tween-20 and then incubated overnight at 4°C with the primary antibodies specific for SRXN1 (Abcam, Cambridge, UK) or KRT6A (Cell Signaling Technology, Danvers, MA, USA). β-Actin (Abcam) was used as the protein loading control. The membranes were then washed with (Tris-buffered saline with 0.1% Tween-20) TBST for 10 min, and incubated for 1 h at room temperature with the appropriate secondary antibody: IRDye 800 CW-conjugated anti-rabbit IgG (Li-Cor, Lincoln, NE, USA). After washing with TBST, the labeled protein bands on the membranes were quantified using an Odyssey infrared Imaging System (Li-Cor).

### RNA Interference

Small interfering RNAs (siRNAs) were designed to knock down the expression of SRXN1 and KRT6A. In this experiment, the siRNA molecules were synthesized by Ribobio Co., Ltd. (Guangzhou, China) as double-stranded RNA oligonucleotides with proprietary chemical modifications. The target sequences of the six siRNAs used to target SRXN1 and KRT6A were as follows:

si-SRXN1-1: GGAGGTGACTACTTCTACT
si-SRXN1-2: CGATGTCCTCTGGATCAAA
si-SRXN1-3: CAGACCTAAGGGTGTACCT
si-KRT6A-1: GAGGAGATTGCTCAGAGAA
si-KRT6A-2: CCAGCAGGAAGAGCTATAA
si-KRT6A-3: TGCCAAGAACAAGCTGGAA

The sense strand of each siRNA is the RNA transcribed from target sequences, and the antisense strand is the complementary RNA of the target sequence. A dTdT sequence was added to the end of each strand to achieve siRNA stability.

Cells were cultured in 6-well plates to 50–60% confluency and then transfected with siRNAs using Lipofectamine^®^ 2000 Reagent (Thermo Fisher, Inc., Chicago, IL, USA) according to the manufacturer’s instructions. Total RNA was isolated from the cells at 48 h post-transfection and subjected to qRT-PCR to measure the efficiency of siRNA-based interference. These cells were also harvested and subjected to western blotting, Cell Counting Kit-8 (CCK-8) assays, flow cytometric analyses and tumorigenicity assays in nude mice.

### CCK-8 Proliferation Assay

Cell proliferation was assessed using CCK-8 (Dojindo Laboratories, Tokyo, Japan) according to the manufacturer’s protocol. In brief, approximately 5.0 × 10^3^ cells were plated into each well of a 96-well plate, followed by incubation for 0, 24, 48 or 72 h. At the indicated time point, 10 μL of CCK-8 solution was added to each well, followed by another 1 h incubation at 37°C. The cell proliferation curves were plotted by measuring the absorbance at 450 nm in each well at each indicated time point. Three wells were used per experimental condition, and all experiments were performed in triplicate.

### Flow Cytometric Analysis

Apoptosis was assessed using the Annexin V-PE/7AAD Apoptosis Detection Kit (KeyGen Biotech, Nanjing, China). Briefly, cells were harvested using EDTA-free trypsin, washed twice with ice-cold phosphate-buffered saline (PBS), collected, and counted. Next, 50 μL of binding buffer and 5 μL of 7-AAD were added to aliquots of 1 × 10^5^ cells in flow cytometry tubes, which were then incubated for 10 min in the dark. Then, 450 μL of Binding Buffer and 1 μL of Annexin V-PE were added to each tube. After a 10 min incubation in the dark, the cells were analyzed on a CytoFLEX Cytometer (Beckman-Coulter, CA, USA).

### Tumorigenicity Assays in Nude Mice

BALB/c nude mice (age, 4–5 weeks; body weight, 20–25 g) were obtained from Guangdong Medical Laboratory Animal Center (Guangzhou, China). All experimental procedures involving animals were in accordance with the Guide for the Care and Use of Laboratory Animals and the institutional ethical guidelines for experiments involving animals. A549 cells in the logarithmic growth phase were collected for inoculation. Mice were randomly divided into 3 groups to test the roles of *SRXN1* and *KRT6A*: control, negative control (NC) and siRNA (3 groups for each gene, giving 6 groups in total; n = 6 per group, including 3 male and 3 female). Briefly, 2.0 × 10^6^ cells were suspended in 200 µL of a 1:1 mixture of Matrigel^®^ Basement Membrane Matrix High Concentration (Corning, NY, USA) and complete medium, and then injected into the right lower groin of each mouse. The length (a) and width (b) of each xenograft tumor were measured every 4 days with a vernier caliper. Tumor volumes (mm^3^) were calculated using the following formula: volume = ab^2^/2. Mice were sacrificed 4 weeks after inoculation by cervical dislocation, and the tumors were excised, weighed and subjected to an immunohistochemical (IHC) analysis.

### IHC Analysis

Histological sections of the tumor xenografts were excised, fixed in 4% paraformaldehyde for 24 h and embedded in paraffin. Subsequently, 5-µm tissue sections were used in a diagnostic examination of tumor pathology. First, the paraffinized sections were dewaxed by washing with xylene, absolute ethyl alcohol, 75% alcohol and distilled water in sequence. Next, hematoxylin–eosin (H&E) staining of some sections was performed to visualize morphological alterations. Other dewaxed sections were put in to microwave oven, BSA seal. After examination under a microscope, the sections were incubated with a primary antibody specific for Ki-67 (dilution, 1:100; Abcam) overnight at 4°C, and subsequently with a secondary antibody labeled with horseradish peroxidase (Beyotime) for 1 h at 37°C. The DAB Horseradish Peroxidase Color Development Kit (Beyotime) and TMB chromogenic substrate were used to enable the peroxidase-catalyzed final brown coloration of the labeled areas.

### Statistical Analysis

All statistical analyses were performed using the SPSS version 25 software package (SPSS, Inc., Chicago, IL, USA). Values are expressed as means ± standard deviations $\left(\bar{x}\pm SD\right)$ . Continuous variables are displayed as means and standard deviations, whereas categorical variables are presented as proportions. In univariate analyses, the qualitative variables were compared using a t-test (or non-parametric test when necessary). The mean values of quantitative variables were compared using Student’s t-test, and differences between the groups were analyzed using an ANOVA. The Shapiro–Wilk and Levene tests were used to test the normality of distribution of the residuals and the homogeneity of variances, respectively. The Wilcoxon–Mann–Whitney test was performed when the basic assumptions of Student’s t-test were not satisfied. A *P*-value <0.05 was considered to indicate a statistically significant difference.

## Results

### Identification of 27 Hub SRGs From Healthy to Lung Cancer in Smokers by Bioinformatics Analysis

**Figure 1:**
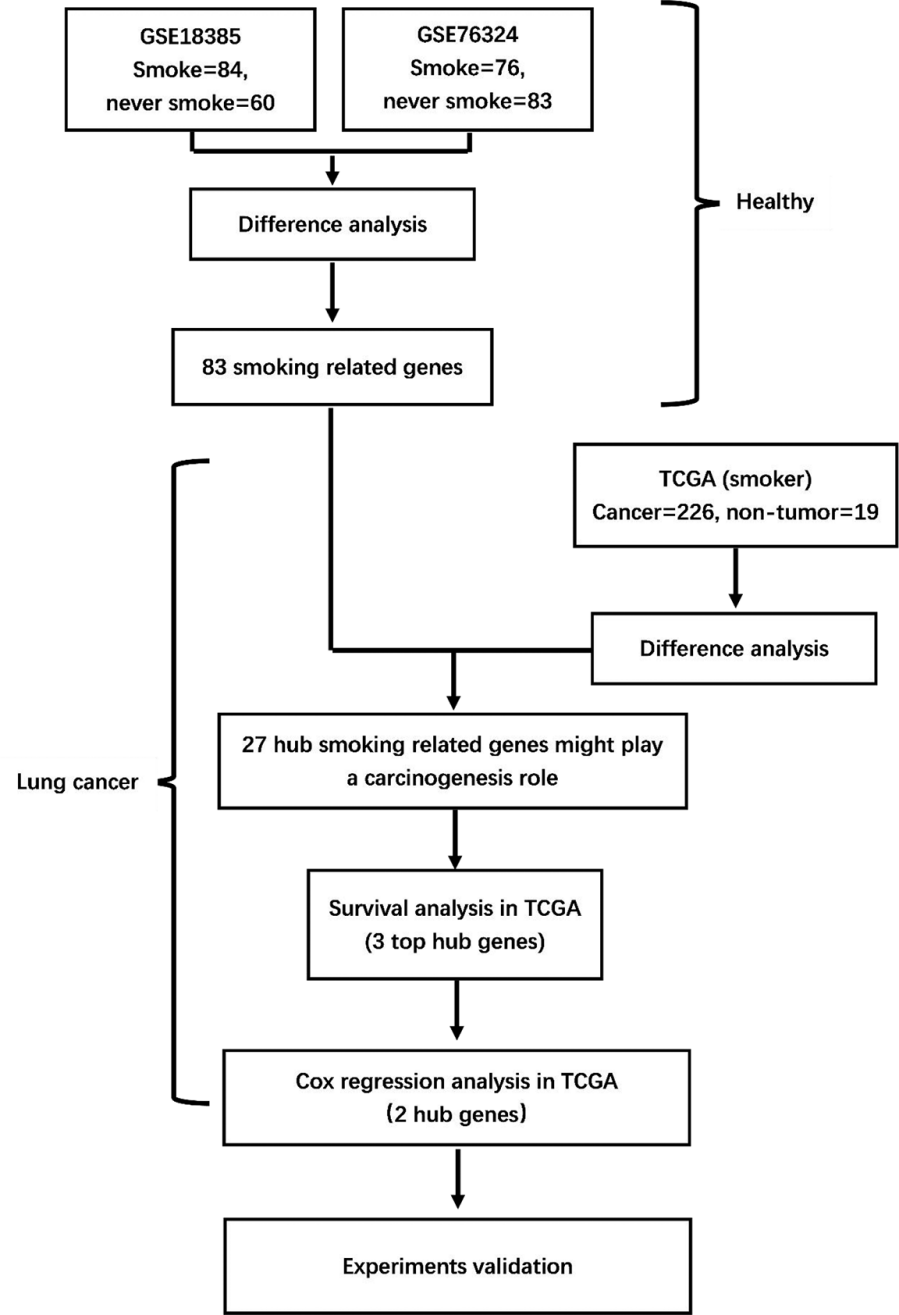
Flowchart of the study. GSE18385 and GSE76324, Gene Expression Omnibus cohorts; TCGA, The Cancer Genome Atlas.

**Figure 2:**
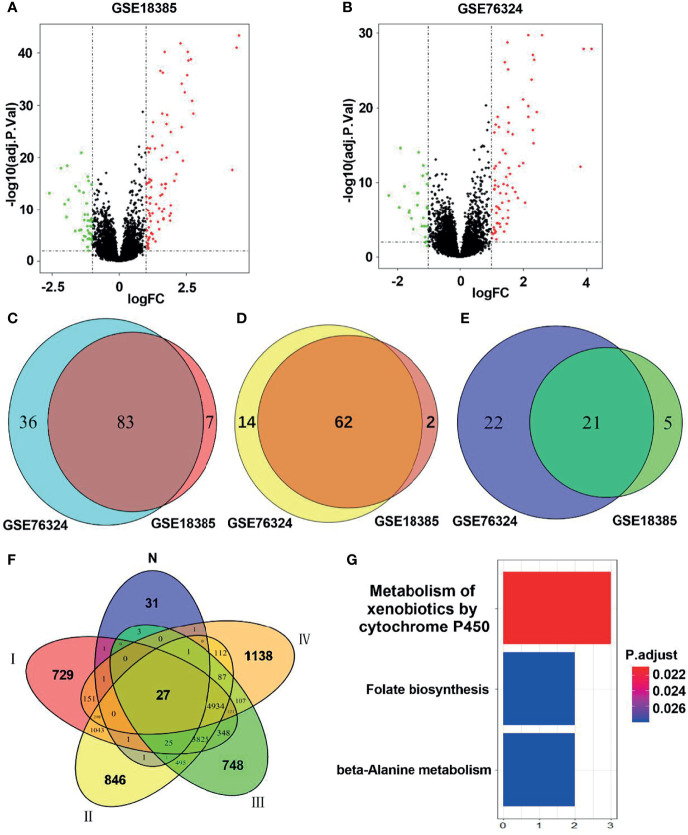
Identification of hub smoking-related genes (SRGs). **(A, B)** Volcano plots of differentially expressed genes in the GSE18385 and GSE76324 cohorts. Log2 (FC) vs. -log10 (adj.P.Val) for differentially expressed genes. Red and blue dots represent upregulated and downregulated genes, respectively (log2|FC| > 1, adj.P.Val < 0.05). **(C)** Venn diagram of SRGs in GSE18385 and GSE76324. **(D)** Venn diagram of upregulated differentially expressed genes in GSE18385 and GSE76324. **(E)** Venn diagram of downregulated differentially expressed genes in GSE18385 and GSE76324. **(F)** Venn diagrams plotted to showcase overlaps between the 83 SRGs and TCGA dataset. N represents 83 SRGs from healthy smokers, while I, II, III and IV represent clinical stages I, II, III and IV among smokers with lung cancer, respectively. **(G)** Significantly enriched Kyoto Encyclopedia of Genes and Genomes pathways corresponding to the 27 hub SRGs.

As shown in the flowchart in **Figure 1**, two training cohorts of healthy human participants, GSE18385 and GSE76324, were downloaded from the GEO database for the differential gene expression analysis. Using the cut-off criteria of an adjusted *P*-value <0.05 and |log2FC| >1, 83 SRGs were detected by overlapping the differential expression profiles of the two cohorts (**Figures 2A–C**). As shown in **Figures 2D, E**, 62 and 21 of these SRGs were upregulated and downregulated, respectively.

To identify hub SRGs that might contribute to lung cancer development, we evaluated the expression of these 83 SRGs in lung cancers, using data from TCGA. We identified potential oncogenes and tumor suppressor genes by calculating the differential gene expression between non-tumor samples and samples of each clinical stage of NSCLC. We identified 27 hub SRGs that were significantly correlated with lung cancer development through our integrated analysis of the 83 SRGs and the differentially expressed genes identified from TCGA (**Figure 2F**). As shown in **Table 2**, 19 of these 27 hub SRGs exhibited upregulated expression in smokers, with further increases observed in smokers with NSCLC, whereas 7 exhibited downregulated expression in smokers and were further reduced in smokers with NSCLC. Only *KLHDC8A* was downregulated by smoking but upregulated in smokers with NSCLC. A KEGG analysis indicated that the 27 hub SRGs were significantly enrichened in the pathways of “metabolism of xenobiotics by cytochrome P450” and “folate biosynthesis and beta-alanine metabolism” (**Figure 2G**). All of these results indicate that these 27 SRGs might be the key genes contributing to smoking-related lung cancer.

**Table 2:** Differential expression levels of 27 hub smoking-related genes between healthy smokers and those with lung cancer.

Gene	Healthy person (GEO)		Smoker (TCGA)			
	Smokers vs. Never-smokers		Cancer vs. Normal			
	GSE18385	GSE76324	Stage-I	Stage-II	Stage-III	Stage-IV
SRXN1	1.13	1.24	2.21	2.30	2.48	1.98
KRT6A	1.15	1.08	7.08	6.89	7.10	6.60
SPP1	1.02	1.27	3.97	3.86	4.41	2.35
AKR1C1	1.08	1.16	3.98	3.62	4.10	3.32
CNGB1	1.10	1.16	2.95	2.08	1.86	2.59
HOXA1	1.13	1.08	2.61	2.35	2.60	2.15
CLDN10	1.20	1.27	2.62	2.79	2.95	3.76
NR0B1	1.26	1.50	7.77	7.11	7.07	7.11
NQO1	1.51	1.69	2.64	2.42	2.27	3.35
CBR1	1.52	1.76	1.71	1.43	1.76	1.78
GAD1	1.55	1.76	3.73	3.46	3.92	3.14
TPRXL	1.83	1.91	5.00	4.74	4.62	5.19
GPX2	2.16	2.29	6.09	6.10	6.18	5.30
UCHL1	2.17	2.33	3.87	3.86	4.12	3.99
CABYR	2.26	2.54	5.27	4.94	4.94	4.94
JAKMIP3	2.32	2.38	3.27	2.98	3.12	3.57
ALDH3A1	2.36	2.57	2.42	1.98	3.41	3.52
SLC7A11	2.43	2.72	3.79	3.65	3.37	2.61
AKR1B10	4.17	4.49	6.78	6.31	6.36	3.78
ITLN1	-2.27	-2.60	-4.88	-4.79	-6.28	-3.49
SEC14L3	-1.90	-1.94	-3.38	-4.09	-3.61	-3.09
MT1M	-1.54	-1.42	-3.35	-3.48	-3.20	-3.34
PPP1R16B	-1.18	-1.34	-1.44	-1.75	-1.66	-2.23
SLIT2	-1.16	-1.16	-2.26	-1.99	-2.09	-1.98
PPBP	-1.03	-1.15	-2.65	-2.52	-4.38	-4.81
DPEP2	-1.02	-1.30	-2.63	-2.93	-3.33	-2.56
KLHDC8A	-1.31	-1.11	1.43	1.87	1.96	1.92

GEO, Gene Expression Omnibus; TCGA, The Cancer Genome Atlas.

### Identification of Top SRGs in Smokers With NSCLC *via* Survival and Cox Regression Analyses

**Figure 3:**
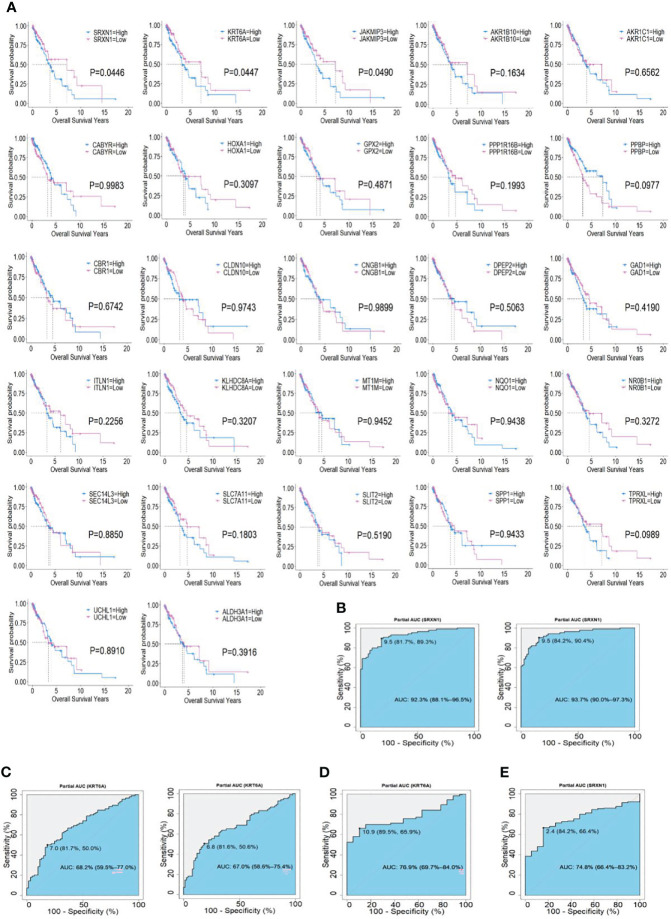
Associations of two top hub smoking-related genes (SRGs) with overall survival in lung cancer patients *via* Kaplan–Meier and Cox regression analyses. **(A)** Kaplan–Meier curves of 27 hub SRGs; **(B)** receiver operating characteristic (ROC) curves of *SRXN1* in the GSE18385 and GSE76324 datasets. **(C)** ROC curves of *KRT6A* in the GSE18385 and GSE76324 datasets. **(D)** ROC curves of *SRXN1* in The Cancer Genome Atlas (TCGA). **(E)** ROC curves of *KRT6A* in TCGA.

To identify the potential prognostic value of these 27 hub SRGs, we evaluated the relationship between the expression of each SRG and overall survival (OS) among smokers with NSCLC. These patients were stratified into high and low expression groups according to the median expression of each SRG. The results of a univariate survival analysis demonstrated that the expression of *SRXN1*, *KRT6A* and *JAKMIP3* was negatively correlated with OS (both *P* < 0.05, **Figure 3A**). The univariate Cox regression analysis revealed that the expression of *SRXN1* (HR = 1.70, *P* < 0.05) and *KRT6A* (HR = 1.71, *P* < 0.05) was significantly associated with the prognosis of patients with NSCLC; no significant association was observed with the expression of *JAKMIP3* (*P* > 0.05, **Table 4**). Moreover, an ROC analysis revealed that *SRXN1* and *KRT6A* were both highly sensitive and specific, suggesting that both had a high diagnostic value for distinguishing smokers from never-smokers (AUC = 92.3% in GSE18385 and 93.7% in GSE76324 for *SRXN1* and 68.2% in GSE18385 and 67.0% in GSE76324 for *KRT6A*, **Figures 3B, C**). Another ROC analysis showed that *SRXN1* and *KRT6A* could clearly distinguish patients with NSCLC from healthy individuals (AUC = 74.8% in TCGA for *SRXN1* and 76.9% in TCGA for *KRT6A*, **Figures 3D, E**). In summary, these results suggest that *SRXN1* and *KRT6A* might be potential biomarkers of smoking exposure and early lung cancer diagnosis and prognosis.

### Upregulation of *SRXN1* and *KRT6A* Expression in Patients With NSCLC and Lung Cancer Cell Lines

**Figure 4:**
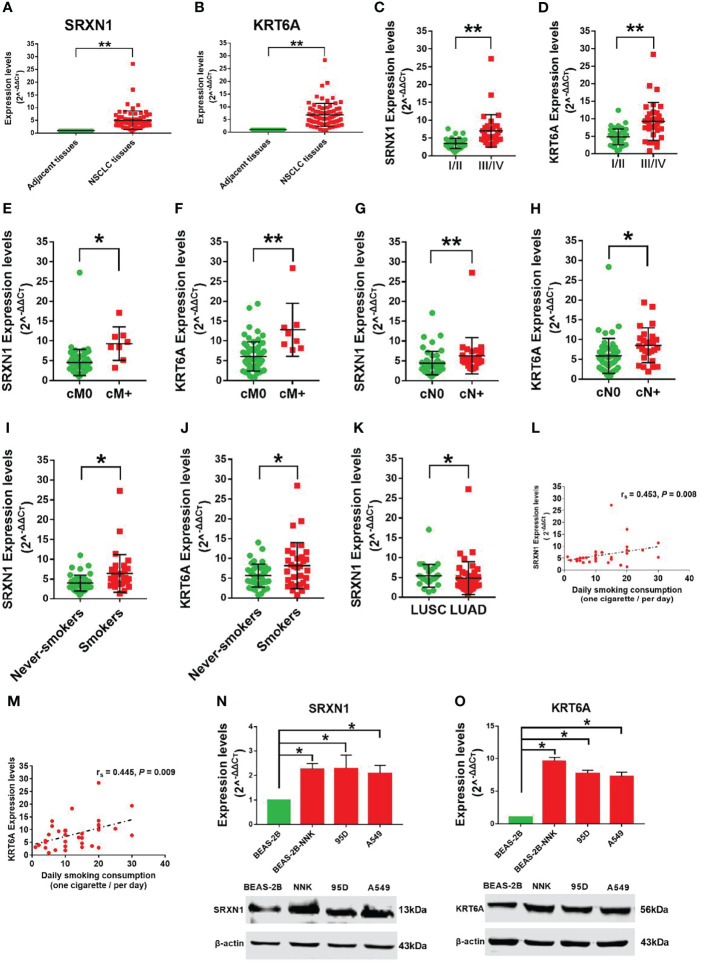
Strong expression of *SRXN1* and *KRT6A* in non-small-cell lung cancer (NSCLC) tissues and lung cancer cell lines. **(A)** *SRXN1* expression was detected in NSCLC and normal tissues by RT-qPCR. **(B)** *KRT6A* expression was detected in NSCLC and normal tissues by RT-qPCR. **(C, D)** Higher expression of *SRXN1* and *KRT6A* in patients with clinical stage III/IV NSCLC vs. those with clinical stage I/II NSCLC. **(E, F)** Higher expression of *SRXN1* and *KRT6A* in patients with cM+ NSCLC vs. patients with cM0 NSCLC. **(G, H)** Higher expression of *SRXN1* and *KRT6A* in patients with cN+ NSCLC vs. patients with cN0 NSCLC. **(I, J)** Higher expression of *SRXN1* and *KRT6A* in smokers with NSCLC vs. never-smokers with NSCLC. **(K)** Higher expression of *SRXN1* in LUAD patients vs. LUSC patients. **(L, M)** Scatter plots of the correlations between *SRXN1* and *KRT6A* expression and daily smoking consumption. **(N, O)** RT-qPCR and western blot analyses of the SRXN1 and KRT6A mRNA and protein levels in 95D, A549 and Beas-2B-NNK cells vs. with Beas-2B cells. *P < 0.05, **P < 0.01.

To verify the carcinogenic roles of *SRXN1* and *KRT6A* in NSCLC, we measured the expression of these genes in 75 paired NSCLC and non-tumor tissues by RT-qPCR. Both *SRXN1* and *KRT6A* were significantly upregulated in NSCLC tissues relative to non-tumor tissues (5.07 ± 3.65 fold and 6.80 ± 4.53 fold, respectively; both *P* < 0.001, **Figures 4A, B**). Correlations were observed between increasing expression levels of *SRXN1* and *KRT6A* and an advanced TNM stage (*P* < 0.001 for both, **Figures 4C, D**), distant metastasis (*P* = 0.001 and *P* < 0.001, respectively, **Figures 4E, F**), lymph node metastasis (*P* < 0.001 and *P* = 0.003, respectively, **Figures 4G, H**) and smoking (*P* = 0.001 and *P* = 0.03, respectively, **Figures 4I, J**). Furthermore, higher *SRXN1* expression levels were observed in squamous patients than in adenocarcinoma patients (*P* < 0.05, **Figure 4K**), suggesting that *SRXN1* might be correlated with the lung cancer subtype. A Spearman rank correlation analysis revealed that the expression levels of *SRXN1* and *KRT6A* were significantly correlated with daily smoking consumption (r = 0.453 and r = 0.445, respectively, both *P* < 0.05, **Figures 4L, M**). However, neither *SRXN1* nor *KRT6A* expression was correlated significantly with age, sex or tumor size (*P* > 0.05, **Table 3**).

**Table 3:** Univariate Cox regression analysis of smoking-related genes (SRGs) in The Cancer Genome Atlas (TCGA).

Gene	*P*-value (log rank)	Beta (Cox)	HR (95% CI)	*P*-value (Cox)
KRT6A	0.045	0.530	1.700 (1.007–2.869)	0.0471
SRXN1	0.045	0.535	1.708, (1.007–2.987)	0.0471
JAKMIP3	0.049	0.513	1.671, (0.997–2.800)	0.0513

CI, confidence interval.

**Table 4:** Non-parametric assessment of the correlations between clinicopathological factors and *SRXN1* and *KRT6A* expression levels in 75 patients with non-small-cell lung cancer.

Characteristics	No. of Patients	*SRXN1*	*P*-value	*KRT6A*	*P*-value
Age (years)					
≤60	35	4.43 ± 2.41	0.234	6.27 ± 3.94	0.410
>60	40	5.62 ± 4.46		6.80 ± 4.57	
Sex					
Male	49	5.18 ± 4.27	0.632	6.63 ± 3.56	0.620
Female	26	4.86 ± 2.23		7.11 ± 6.10	
Smoking history					
Smoker	33	6.42 ± 4.76	0.001	8.19 ± 5.82	0.030
Never-smoker	42	4.00 ± 2.03		5.70 ± 2.89	
Histology					
Squamous	30	5.41 ± 2.87	0.031	6.62 ± 4.05	0.837
Adenocarcinoma	45	4.84 ± 4.14		6.92 ± 4.92	
cTNM stage					
І/П	41	3.48 ± 1.42	< 0.001	4.79 ± 2.30	<0.001
Ш/IV	34	6.98 ± 4.58		9.22 ± 5.41	
cN stage					
cN0	49	4.42 ± 2.96	< 0.001	5.87 ± 4.42	0.003
cN+	26	6.28 ± 4.56		8.55 ± 4.40	
cM stage					
cM0	67	4.56 ± 3.30	0.001	6.08 ± 3.70	<0.001
cM+	8	9.30 ± 4.21		12.83 ± 6.70	
Tumor sizes					
≤3.5	35	4.85 ± 3.12	0.663	6.92 ± 5.45	0.663
>3.5	40	5.25 ± 4.14		6.69 ± 3.70	

cTNM, clinical tumor–node–metastasis stage; cN, clinical nodal stage; cM, clinical metastasis stage.

Furthermore, we observed increased expression of SRXN1 and KRT6A mRNA and protein in lung cancer cell lines (95D, A549) and BEAS-2B-NNK cells, compared with normal BEAS-2B cells (both *P* < 0.05, **Figures 4N, O**). These results imply that SRXN1 and KRT6A might play a role in the carcinogenesis of NSCLC and are closely related to smoking.

### Knockdown of *SRXN1* or *KRT6A* Inhibited the Viability and Promoted the Apoptosis of Lung Cancer Cells

**Figure 5:**
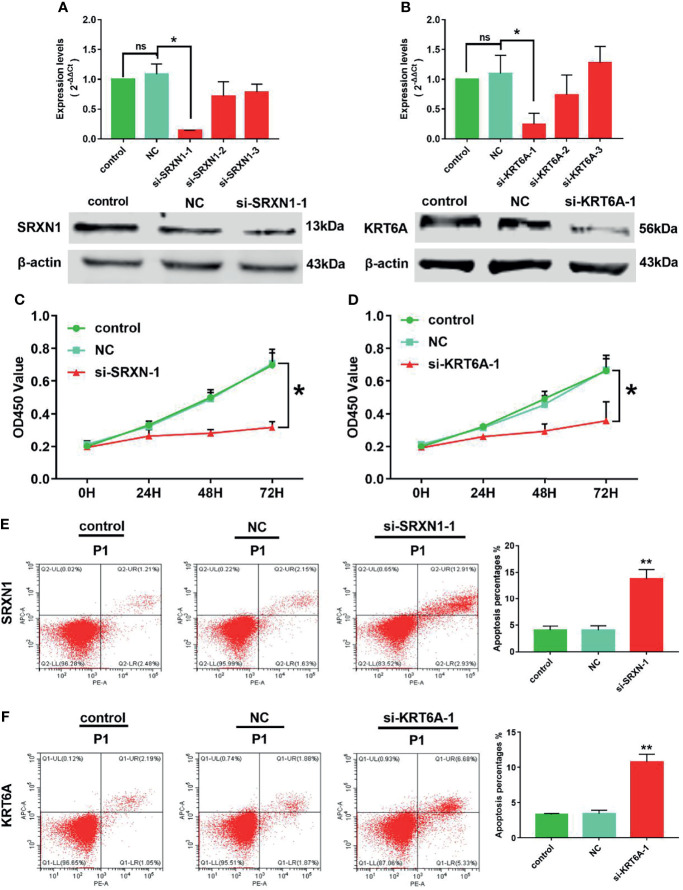
*SRXN1* or *KRT6A* inhibition suppressed cell viability and promoted cell apoptosis. **(A)** RT-qPCR and western blot analyses were performed to test the effect of *SRXN1* interference. **(B)** RT-qPCR and western blot analyses were performed to test the effect of *KRT6A* interference. **(C)** Cell viability in response to *SRXN1* depletion was monitored using a CCK-8 assay. **(D)** Cell viability in response to *KRT6A* depletion was monitored using a CCK-8 assay. **(E)** Representative flow cytometry plots of cell apoptosis in response to *SRXN1* depletion and a graph of apoptosis rates per group. **(F)** Representative flow cytometry plots of cell apoptosis in response to *KRT6A* depletion and a graph of apoptosis rates per group. *P < 0.05, **P < 0.01. NC, negative control; ns, no significance.

Because SRXN1 and KRT6A expression was upregulated in lung cancers, we designed three siRNAs to suppress the endogenous expression of *SRXN1* and *KRT6A* transcripts. Both RT-qPCR and western blotting were used to examine the effectiveness of siRNA interference. The levels of SRXN1 or KRT6A mRNAs and proteins were obviously decreased in A549 cells after transfection with si-*SRXN1*-1 or si-*KRT6A*-1, respectively, in comparison with the NC and control groups. However, si-*SRXN1*-2, si-*SRXN1*-3, si-*KRT6A*-2 and si-*KRT6A*-3 had no significant effects on *SRXN1* or *KRT6A* expression (**Figures 5A, B**). Therefore, si-*SRXN1*-1 and si-*KRT6A*-1 were selected for use in subsequent experiments.

CCK-8 proliferation assays were performed to assess the viability of the cells subjected to knockdown. We observed a significant reduction in the viability of A549 cells subjected to *SRXN1* or *KRT6A* silencing, compared with the NC and control groups, respectively. At 72 h after siRNA transfection, the cell viability in the si-*SRXN1*-1 and si-*KRT6A*-1 groups were reduced by 54.5% (*P* < 0.05, **Figure 5C**) and 46.1% (*P* < 0.05, **Figure 5D**), respectively. A flow cytometry analysis revealed that A549 cells exhibited enhanced apoptosis under *SRXN1* or *KRT6A* suppression (both *P* < 0.05, **Figures 5E, F**), compared with the NC and control groups.

### *SRXN1* or *KRT6A* Knockdown Inhibited Tumor Growth *In Vivo*

**Figure 6:**
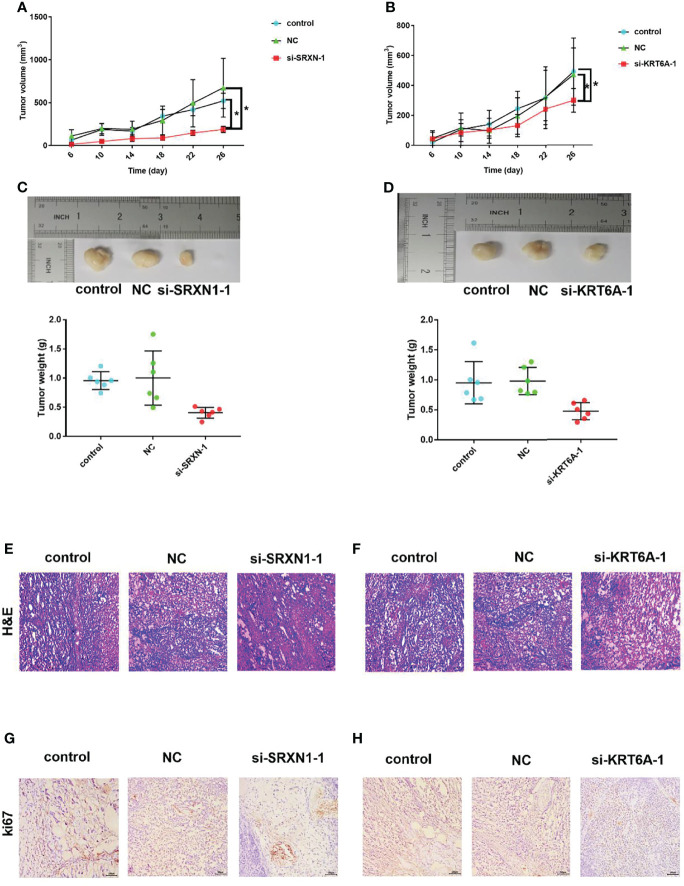
*SRXN1* or *KRT6A* knockdown suppressed tumor growth *in vivo*. **(A)** Tumor growth curve in response to *SRXN1* depletion. **(B)** Tumor growth curve in response to *KRT6A* depletion. **(C)** Representative images of xenograft tumors from nude mice and comparison of tumor weights in response to *SRXN1* depletion. **(D)** Representative images of xenograft tumors from nude mice and comparison of tumor weights in response to *KRT6A* depletion. **(E, F)** Representative images of hematoxylin–eosin-stained tumor samples from *SRXN1* and *KRT6A* knockdown samples, respectively, relative to controls and negative controls (NC). **(G, H)** Representative images of Ki67-immunostained tumor samples from *SRXN1* and *KRT6A* knockdown samples, respectively, relative to controls and NCs. *P < 0.05.

The significant effects of *SRXN1* or *KRT6A* knockdown on cell growth *in vitro* warranted an exploration of the roles of *SRXN1* and *KRT6A in vivo*. Nude mice xenograft models were generated by the subcutaneous injection of si-*SRXN1*- or si-*KRT6A*-transfected A549 cells, siRNA NC-transfected A549 cells or control A549 cells. The tumor growth curves indicated significant growth inhibition in the si-*SRXN1*-1 and si-*KRT6A*-1 groups (both *P* < 0.05, **Figures 6A, B**), compared with the NC and control groups, respectively. The tumor volumes and weights in the si-*SRXN1*-1 and si-*KRT6A*-1 groups were obviously reduced relative to those in the NC and control groups (both *P* < 0.05), but there was no difference between the NC and control groups (**Figures 6C, D**).

To delineate the underlying mechanism, the harvested tumors were further subjected to H&E staining and IHC analysis. H&E staining of the xenograft tumors revealed spherical cells with large, deeply stained nuclei (**Figures 6E, F**). The average percentages of Ki67-positive cells in the si-*SRXN1*-1 and si-*KRT6A*-1 groups were significantly lower than those in the NC and control groups (**Figures 6G, H**). This suppressive effect of *SRXN1* or *KRT6A* knockdown on tumor growth was consistent with the results *in vitro*.

## Discussion

Cigarette smoking is the most common etiology of NSCLC, accounting for the vast majority of cases worldwide (31), and remains one of the strongest risk factors for NSCLC occurrence and development. Additionally, a large majority of lung cancer deaths are attributable to cigarette smoking. Cigarettes contain a complex mixture of components and are associated with complex molecular mechanisms of carcinogenesis (32, 33). It is therefore imperative to curb the rates of cigarette smoking. Developments in gene sequencing technology have enabled the identification of some potential gene markers with predictive value in cigarette smokers and patients with NSCLC (34, 35). However, few reliable markers are available. Therefore, the identification of reliable biomarkers that more accurately predict the early diagnosis and prognosis of NSCLC is urgently needed.

Significant developments in high-throughput sequencing technology have led more researchers to devote close attention to bioinformatics as a means of identifying the key genes in cigarette-related carcinogenesis. For instance, Wang et al. (36) identified three smoking-related genes, namely *GYPC* (glycophorin C), *NME1* (NME/NM23 nucleoside diphosphate kinase 1) and *SLIT2* (slit guidance ligand 2), that were significantly associated with cigarette smoke-induced LUAD, based on an integration analysis of four GEO datasets and an mRNA sequencing analysis. Ren et al. (37) reported that the expression of nine key genes, namely *UBE2T*, *EXO1*, *TOP2A*, *CDCA7*, *HMMR*, *ANLN*, *RAD54L*, *DEPDC1* and *CDCA8*, was correlated with an adverse prognosis in patients with smoking-related LUAD. Moreover, Huang et al. (38) identified several candidate microRNAs that might be useful for assessing the risk of smoking-related lung diseases based on an integrated analysis of four GEO datasets. Chawsheen et al. (39) reported that lung cancer patients with high sulfiredoxin level was associated with a significantly shorter survival duration in a bioinformatics analysis of lung cancer patients. Sarill et al. (40) reported that exposure to cigarette smoke extract induced a significant increase in the expression of *SRXN1* mRNA. Xiao et al. (41) observed that high *KRT6A* expression was correlated with an unfavorable prognosis in a bioinformatics analysis of lung adenocarcinoma patients. These previous studies confirm the important roles of *SRXN1* and *KRT6A* in lung cancer. Consistently, we identified both genes as the top two SRGs with potential carcinogenic effects and high predictive values with respect to smoking exposure and the early diagnosis and prognosis of NSCLC through our bioinformatics analyses.

*SRXN1*, an endogenous antioxidant, has been shown to protect against exogenous compound-induced oxidative stress *in vitro* and *in vivo* (42, 43). However, numerous studies reported that the anti-oxidative stress effect of *SRXN1* could not protect against pulmonary disease (44). Jiang et al. (45) reported that SRXN1 promoted colorectal cancer cell invasion and metastasis by enhancing EGFR signaling. *KRT6A*, a member of the keratin protein family, plays an important role in squamous epithelial epidermalization. Besides, cancer-related studies identified associations between the expression of *KRT6A* and several diseases and cancers, such as pachyonychia congenita, oral squamous cell carcinoma, lung cancer, renal carcinoma and progressive breast cancer (46–48). In one study, *KRT6A* silencing suppressed nasopharyngeal carcinoma cell invasion and metastasis *via* the β−catenin cascade (49).

Cigarette smoking, which is preventable, remains the main risk factor for lung cancer worldwide (50). Changes in gene expression in response to environmental contact may signal exposure to toxins. To identify genes with altered expression levels in response to cigarette smoking, we compared the transcriptomes of lung tissues from smokers and never-smokers from two GEO datasets and a TCGA dataset using a bioinformatics analysis. We found that *SRXN1* and *KRT6A* were differentially expressed between both groups in all three datasets, with concordant higher expression in ever-smokers. Meanwhile, we observed the smoking-induced upregulation of *SRXN1* and *KRT6A* expression in 75 matched tumor–normal tissue pairs from patients with NSCLC who were enrolled in our study. More importantly, both SRXN1 and KRT6A expression were significantly correlated with smoking so that we proposed an assumption that SRXN1 and KRT6A might be the key for preventing smoke cause lung cancer. In summary, the findings suggested that *SRXN1* and *KRT6A* might be potential biomarkers of the extent of exposure to environmental tobacco smoke. We believed that SRXN1 and KRT6A might be the early diagnostic biomarkers and therapy targets on clinical therapy of smoke cause lung cancer with further validated study in future.

## Conclusions

In conclusion, *SRXN1* and *KRT6A* expression might be potential biomarkers of smoking exposure and the early diagnosis and prognosis of NSCLC. Our findings shed light on the novel molecular mechanisms underlying the pathophysiology of smoking-related lung cancer, and reveal new pathways that might be therapeutically exploitable.

## Data Availability Statement

Publicly available datasets were analyzed in this study. This data can be found here: https://www.ncbi.nlm.nih.gov/geo/query/acc.cgi GEO database: GSE18385, GSE76324. The data of TCGA can be found in TCGA database by the following link: https://portal.gdc.cancer.gov/repository.

## Ethics Statement

The studies involving human participants were reviewed and approved by the Ethics Committee of Guangzhou medical university. The patients/participants provided their written informed consent to participate in this study. The animal study was reviewed and approved by the Ethics Committee of Guangzhou medical university. Written informed consent was obtained from the owners for the participation of their animals in this study. Written informed consent was obtained from the individual(s) for the publication of any potentially identifiable images or data included in this article.

## Author Contributions

QY was responsible for the concept. QY was responsible for funding acquisition. QY designed the experiments. JZZ, GJ, and EX collected and analyzed data. JXZ and LL did data interpretation and discussions. JZZ and GJ initially drafted the paper. All co-authors reviewed and edited the manuscript and approved the submission.

## Funding

This work was supported by the National Natural Science Foundation of China (Grant Number: 81773385 and 81472937 to QY), Natural Science Foundation of Guangdong Province (Grant Number: 2019A1515011298 to QY).

## Conflict of Interest

The authors declare that the research was conducted in the absence of any commercial or financial relationships that could be construed as a potential conflict of interest.

## Publisher’s Note

All claims expressed in this article are solely those of the authors and do not necessarily represent those of their affiliated organizations, or those of the publisher, the editors and the reviewers. Any product that may be evaluated in this article, or claim that may be made by its manufacturer, is not guaranteed or endorsed by the publisher.
