# Supplementary material for: Identification of SRXN1 and KRT6A as Key Genes in Smoking-Related Non-Small-Cell Lung Cancer Through Bioinformatics and Functional Analyses
Source: Front Oncol. 2022 Jan 5;11:810301. doi: 10.3389/fonc.2021.810301 (PMC8767109; doi:10.3389/fonc.2021.810301)
Supplement: Supplementary file 1 [file Table_1.docx]

**Table S1.** Characteristics of the NSCLC smokers in TCGA (n or mean ± SD).

| **Characteristics** | **Tumor sample**  **(n = 226 )** | **Normal sample**  **(n = 19 )** |
| --- | --- | --- |
| Age, years | 62.90 ± 9.75 | 66.79 ± 7.17 |
| Number pack years smoked | 52.60 ± 27.28 | 63.22 ± 32.29 |
| Gender, n (%) | | |
| Male | 158 (69.9) | 15 (78.9) |
| Female | 68 (30.1) | 4 (21.1) |
| Race, n (%) | | |
| White | 173 (76.5) | 16 (84.2) |
| Black or African American | 22 (9.7) | 1 (5.3) |
| Asian | 3 (1.3) | 0 |
| Diagnosis, n (%) | | |
| Lung Adenocarcinoma | 104 (46.0) | 7 (36.8) |
| Lung Squamous Cell Carcinoma | 122 (54.0) | 12 (63.2) |
| Event, n (%) | | |
| Alive | 164 (72.6) | 13 (68.4) |
| Dead | 62 (27.4) | 6 (31.6) |
| Stage, n (%) | | |
| I | 113 (50) | 12 (63.2) |
| II | 70 (31) | 4 (21.1) |
| III | 36 (15.9) | 1 (5.3) |
| IV | 7 (3.1) | 2 (10.5) |

**Table S2.** Clinical and characteristics of study patients from Asian.

| **Characteristics** | **N=75** | |
| --- | --- | --- |
| Age (Mean±SD) | 58.65 ± 12.03 | |
| Daily smoking consumption (Mean±SD) | 5.61 ± 8.09 | |
| Tumor Size (Mean±SD) | 3.84 ± 1.37 | |
| Sex (n, %) |  |  |
| Male | 49 (65.33) | |
| Female | 26 (34.67) | |
| Histological Typing (n, %) |  |  |
| Squamous | 30 (40.00) | |
| Adenocarcinoma | 45 (60.00) | |
| Smoking history (n, %) |  | |
| Smoker | 33 (44.00) | |
| Never-smoker | 42 (56.00) | |
| TMN Stage (n, %) |  |  |
| I | 2 (2.67) | |
| II | 39 (52.00) | |
| III | 27 (36.00) | |
| IV | 7 (9.33) | |
| Lympho Node Metastasis (n, %) |  |  |
| Yes | 26 (34.67) | |
| No | 49 (65.33) | |
| Distant Metastasis (n, %) |  |  |
| Yes | 8 (10.67) | |
| No | 67 (89.33) | |
